# Instagram Posts Related to Backwoods Cigarillo Blunts: Content Analysis

**DOI:** 10.2196/22946

**Published:** 2021-02-09

**Authors:** Stephanie Kim, Alia Mourali, Jon-Patrick Allem, Jennifer B Unger, Tess Boley Cruz, Sabrina L Smiley

**Affiliations:** 1 Department of Preventive Medicine Keck School of Medicine University of Southern California Los Angeles, CA United States

**Keywords:** Instagram, blunts, Backwoods cigarillos, smoking

## Abstract

**Background:**

Instagram, one of the most popular social media platforms among youth, offers a unique opportunity to examine blunts—partially or fully hollowed-out large cigars, little cigars, and cigarillos that are filled with marijuana. Cigarillo brands like Backwoods (Imperial Tobacco Group Brands LLC) have product features that facilitate blunt making, including a variety of brand-specific flavors that enhance the smoking experience (eg, honey, dark stout). Backwoods has an active online presence with a user-friendly website.

**Objective:**

This study examined the extent to which Backwoods cigarillo–related posts on Instagram showed blunt making. Instagram offers a unique opportunity to examine blunt making as Instagram accounts will contain images reflective of behavior occurring without the prime of a researcher.

**Methods:**

Data consisted of publicly available Instagram posts with the hashtag #backwoods collected from August 30 to September 12, 2018. Inclusion criteria for this study included an Instagram post with the hashtag “#backwoods”. Rules were established to content analyze posts. Categories included *Type of post* (ie, photo, video, or both); *Blunt-related hashtags* (ie, the corresponding post caption contained one or more hashtags like #blunts, #cannabis, and #weed that were identified in previous social media research); *Rolling blunts* (ie, the post contained an image of one or more individuals rolling a Backwoods cigarillo visibly containing marijuana); and *Smoking blunts* (ie, the post contained an image of one or more individuals blowing smoke or holding a lit blunt). We coded images for *Product flavor* reference, where a code of 1 showed a Backwoods cigarillo pack with a brand-specific flavor (eg, honey, dark stout, Russian crème) visible in the blunt-related image, and a code of 0 indicated that it was not visible anywhere in the image.

**Results:**

Among all posts (N=1206), 871 (72.2%) were coded as *Blunt-related hashtags*. A total of 125 (10.4%) images were coded as *Smoking blunts*, and 25 (2.1%) were coded as *Rolling blunts* (ie, Backwoods cigarillo explicitly used to roll blunts). Among blunt images, 434 of 836 (51.9%) were coded as *Product flavor* (ie, a Backwoods pack with a brand-specific flavor was visible).

**Conclusions:**

Most Backwoods cigarillo–related Instagram images were blunt-related, and these blunt-related images showed Backwoods packages indicating flavor preference. Continued monitoring and surveillance of blunt-related posts on Instagram is needed to inform policies and interventions that reduce the risk that youth may experiment with blunts. Specific policies could include restrictions on product features (eg, flavors, perforated lines, attractive resealable foil pouches, sale as singles) that facilitate blunt making.

## Introduction

Blunts are partially or fully hollowed-out cigars, including little cigars or cigarillos, that are refilled with marijuana. Blunts are an increasingly popular way to smoke marijuana [1,2] and are associated with escalation in use of nicotine and marijuana among adolescents and adults [3-5]. Among US adolescents who have ever used a cigarillo or little cigar, 40% used them to make blunts [6]. Blunt users are exposed to nicotine through the tobacco wrap [7,8], and they are exposed to greater carbon monoxide compared to non–blunt users who smoke marijuana wrapped in a cigarette rolling paper that does not contain nicotine [9,10].

Cigarillos contain just as much nicotine and carcinogens as cigarette smoke, if not more [11,12], leading to increased health risks [13]. Youth and young adults in the United States have among the highest prevalence of cigarillo use [14]. Cigarillos also face fewer federal restrictions (eg, allowed in flavors, sold as singles and in packs of two) than cigarettes [14], but are similar to cigarettes in size, shape, and combustible use [12,14]. Cigarillos are widely available [15-18], and popular brands like Swisher Sweets (Swisher International, Inc) and Backwoods (Imperial Tobacco Group Brands LLC) have features that facilitate blunt making, including perforated lines or tobacco wrappers that are easy to unroll and fill with marijuana, smell-proof resealable foil pouches to conceal marijuana, and availability in a variety of flavors that enhance the smoking experience (eg, honey bourbon, sweet aromatic). Backwoods, in particular, uses advertisement claims that are misleading, like “always true” [19].

Prior research has investigated Backwoods-related posts on Instagram and found marijuana was a common theme [20]. However, it was not determined whether Backwoods cigarillos were being used to complement marijuana use (ie, dual use) or explicitly used to roll blunts. As such, this study is an initial step to examine the extent to which Backwoods cigarillo–related posts on Instagram showed blunt making. Instagram is an image-based platform that has been used to study health-related attitudes and behaviors as well as promotional material from companies [20,21]. Instagram offers a unique opportunity to examine blunt making, as Instagram accounts will contain images reflective of behavior occurring without the prime of a researcher [20,21]. Instagram also offers multicontextual content (images and text) that has provided useful insights about user experiences with tobacco products [20,21]. This is important because blunt use has adverse health effects, and these product features might be expanding the population of tobacco users from marijuana users who otherwise would not use any tobacco.

## Methods

Data consisted of publicly available Instagram posts with the hashtag #backwoods collected from August 30 to September 12, 2018. Netlytic, an Instagram-approved vendor that accessed the public application programming interface of Instagram, was used to collect data. A total of 12,306 posts included the hashtag #backwoods during the study period. Similar to prior Instagram studies [20,21], we numbered each observation and then randomly drew observations using a random number generator until 10% of the sample was culled from the initial corpus. Multiple posts from the same users in either the overall sample frame or in the randomly selected posts were not treated as independent observations. Backwoods (Imperial Tobacco Group Brands LLC) was not the source of any of the posts. Rules were established to content analyze 1206 posts.

The first and second authors generated a codebook based on prior research [20-22] and reviewed a subsample (N=200) of the posts to identify prominent themes. The unit of analysis was the individual Instagram post (ie, the image and corresponding caption), and the coding strategy assessed themes found in the posts. The coding strategy included (1) *Type of post* (ie, photo, video, or both); (2) *Blunt-related hashtags* (ie, the corresponding post caption contained one or more hashtags like #blunts, #cannabis, and #weed that were identified in previous social media research [23,24]); (3) *Rolling blunts* (ie, the post contained an image of one or more individuals rolling a Backwoods cigar visibly containing marijuana); and (4) *Smoking blunts* (ie, the post contained an image of one or more individuals blowing smoke or holding a lit blunt). Similar to previous research using Instagram data [21], we coded images for (5) *Product flavor* reference, where a code of 1 showed a Backwoods package with a brand-specific flavor (eg, honey, dark stout, Russian crème) visible in the image (eg, next to a Backwoods cigarillo that contained marijuana), and a code of 0 indicated that a Backwoods pack with a brand-specific flavor was not visible anywhere in the image. Two investigators (SK and AM) independently coded all posts, and percentage agreement was substantial at 97.0% (*Type of post*; 1170/1206), 90.0% (*Blunt-related hashtags*; 784/871), 100% (*Rolling blunts*; 25/25), 99.2% (*Smoking blunts*; 124/125), and 100% (*Product flavor*; 434/434). Discrepancies were resolved via in-person discussion. We report the percentages of posts for each theme.

## Results

**Figure 1:**
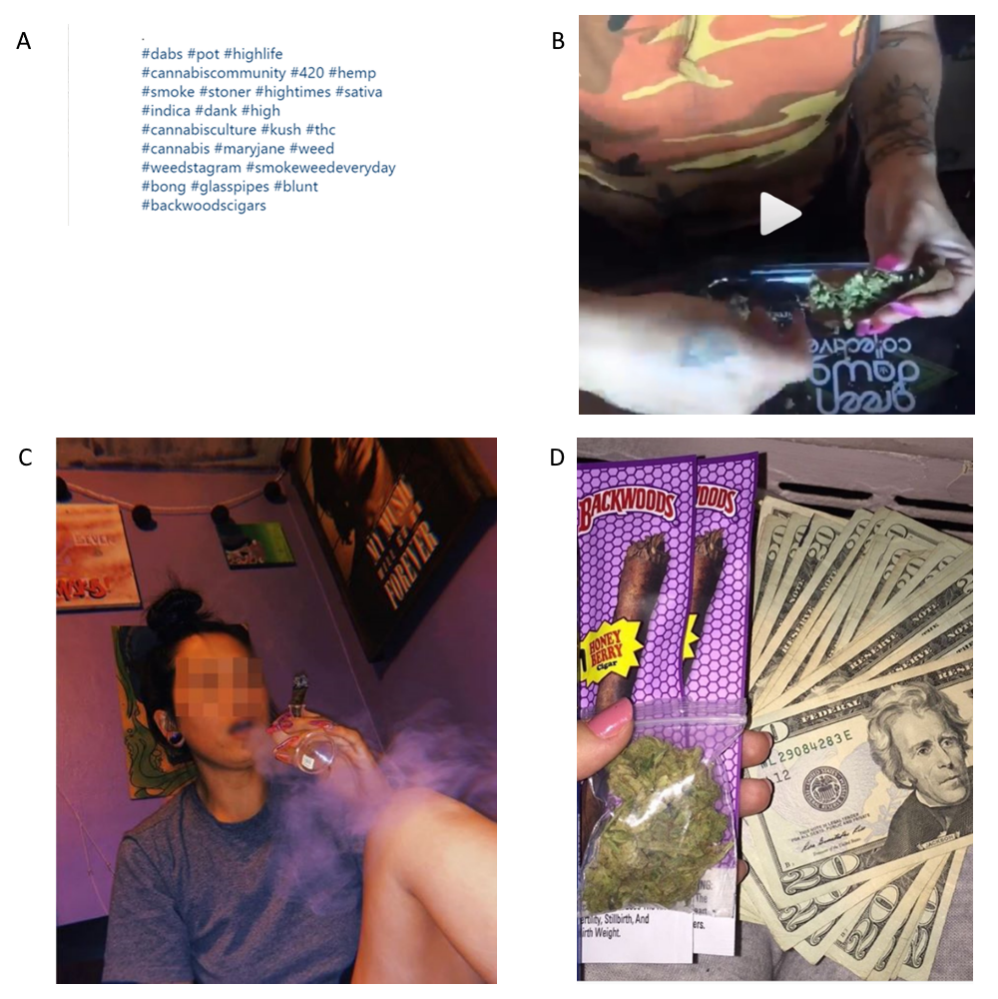
Images representative of select themes: (A) Blunt-related hashtags; (B) Rolling blunts; (C) Smoking blunts; (D) Product flavor.

Among all posts (N=1206), 913 (75.7%) were photos, 268 (22.2%) were videos, and 25 (2.1%) included both photos and videos. A total of 774 (64.2%) were posts from individual Instagram users, and 432 (35.8%) were posts from online tobacco retailers. A total of 871 (72.2%) were coded as *Blunt-related hashtags* (Figure 1A). A total of 125 (10.4%) images were coded as *Smoking blunts* (Figure 1C), and 25 (2.1%) were coded as *Rolling blunts* (ie, Backwoods cigarillo explicitly used to roll blunts; Figure 1B). Among blunt images, 434 of 836 (51.9%) were coded as *Product flavor* (ie, a Backwoods pack with a brand-specific flavor, such as honey, dark stout, or Russian crème, was visible; Figure 1D).

## Discussion

Findings from this study suggest that blunt users perceive Backwoods cigarillos as vehicles for marijuana consumption and that they are comfortable sharing images of this behavior on Instagram. This may be the start of a growing trend as Imperial Brands, the parent company of Imperial Tobacco Group Brands LLC (America’s third-largest tobacco company [25]), recently signed a US $123 million investment deal with Auxly Cannabis Group Inc of Vancouver, Canada [26]. It appears that marijuana may play a substantial role in the tobacco market of the future, especially given that Imperial’s most popular machine-made cigar brand is Backwoods [27].

When considering Backwoods online presence, it should be noted that the age verification on the brand’s website currently reads, “To roll with us, you’ve got to be 21 or over” [19]. This is concerning because Backwoods is clearly aligning itself with blunt making with a website greeting that suggests consumers should consider their tobacco products as good choices for rolling blunts. Findings complement previous research [5,18,19] that cigarillo product features (eg, Backwoods brand name, “natural” leaf wrappers, flavors) facilitate blunt making, including on Instagram. 

Findings suggest that images on Instagram capture the social context in which individuals increasingly display blunt making. Instagram is one of the most popular social media platforms among youth; thus, they could view these public blunt-making posts. Research is needed to examine how youth might be turning to Instagram to learn about blunt making. This includes assessing whether posts were somehow more targeted to youth. Research is also needed to compare the reactions and responses of followers to the posts from individual Instagram users and online tobacco retailers across categories (ie, *Rolling blunts*, *Smoking blunts*). Additionally, blunt making facilitation and use on Instagram should be considered when designing smoking prevention programs for youth.

Findings from this study should be considered with several limitations in mind, including the sole focus on the cigarillo brand Backwoods and related images on Instagram. Findings may not generalize to other companies or social media platforms (eg, Twitter, Facebook, Tumblr). The images analyzed in this study were collected from a 2-week time period and may not generalize to other time periods. Future research should examine longer timeframes, different social media platforms, and additional brands to fully characterize the blunt making and use experience.

This study demonstrated that more than half of Backwoods cigarillo–related Instagram images were blunt-related, and over half of these blunt-related images showed Backwoods packages indicating flavor preference. This study also found that consumers of Backwoods cigarillos were using Instagram to promote smoking blunts and the blunt-making process (eg, rolling). Findings inform the Food and Drug Administration’s regulation of cigar products that are covered under the 2016 Final Deeming Rule [28]. Specific regulations could include restrictions on product features (eg, flavors, perforated lines, attractive resealable foil pouches, sale as singles) that facilitate blunt making.

The University of Southern California Institutional Review Board approved all study procedures.
